# Response to Rhinovirus Infection by Human Airway Epithelial Cells and Peripheral Blood Mononuclear Cells in an In Vitro Two-Chamber Tissue Culture System

**DOI:** 10.1371/journal.pone.0066600

**Published:** 2013-06-17

**Authors:** Devi Rajan, Kelsey A. Gaston, Courtney E. McCracken, Dean D. Erdman, Larry J. Anderson

**Affiliations:** 1 Emory University Department of Pediatrics and Children’s Healthcare of Atlanta, Atlanta, Georgia, United States of America; 2 Division of Viral Diseases, National Center for Immunization and Respiratory Disease, Centers for Disease Control and Prevention, Atlanta, Georgia, United States of America; University Medical Center Freiburg, Germany

## Abstract

Human rhinovirus (HRV) infections are associated with the common cold, occasionally with more serious lower respiratory tract illnesses, and frequently with asthma exacerbations. The clinical features of HRV infection and its association with asthma exacerbation suggest that some HRV disease results from virus-induced host immune responses to infection. To study the HRV-infection-induced host responses and the contribution of these responses to disease, we have developed an in vitro model of HRV infection of human airway epithelial cells (Calu-3 cells) and subsequent exposure of human peripheral blood mononuclear cells (PBMCs) to these infected cells in a two-chamber trans-well tissue culture system. Using this model, we studied HRV 14 (species B) and HRV 16 (species A) induced cytokine and chemokine responses with PBMCs from four healthy adults. Infection of Calu-3 cells with either virus induced HRV-associated increases in FGF-Basic, IL-15, IL-6, IL-28A, ENA-78 and IP-10. The addition of PBMCs to HRV 14-infected cells gave significant increases in MIP-1β, IL-28A, MCP-2, and IFN-α as compared with mock-infected cells. Interestingly, ENA-78 levels were reduced in HRV 14 infected cells that were exposed to PBMCs. Addition of PBMCs to HRV 16-infected cells did not induce MIP-1β, IL-28A and IFN-α efficiently nor did it decrease ENA-78 levels. Our results demonstrate a clear difference between HRV 14 and HRV 16 and the source of PBMCs, in up or down regulation of several cytokines including those that are linked to airway inflammation. Such differences might be one of the reasons for variation in disease associated with different HRV species including variation in their link to asthma exacerbations as suggested by other studies. Further study of immune responses associated with different HRVs and PBMCs from different patient groups, and the mechanisms leading to these differences, should help characterize pathogenesis of HRV disease and generate novel approaches to its treatment.

## Introduction

Human rhinoviruses (HRVs) belong to the genus *Enterovirus* in the family *Picornaviridae*. They are small, non-enveloped, positive strand RNA viruses with a diameter of about 30 nm. There are well over 100 serotypes (or genotypes) of HRVs, phylogenetically divided into three species, A, B, and C. Species A and B viruses can be divided into two receptor binding groups, major and minor. The major group uses inter-cellular adhesion molecule 1 (ICAM1) or CD54 as its receptor (90% of species A and B HRVs) and the minor group uses low-density lipoprotein as its receptor [1], [2], [3]. The receptor for species C HRVs has not yet been identified, but is thought to be distinct. HRVs are the predominant cause of common cold. However, HRV infection also causes lower respiratory tract disease including bronchitis, bronchiolitis and pneumonia [4], and is associated with wheezing and asthma exacerbations in children and adults [5], [6], [7]. These studies demonstrate that HRV infection is commonly associated with asthma exacerbations, i.e. detected in 50% to 80% of patients with exacerbations, and it is likely that the HRV-induced host response to infection is important to these exacerbations. Hence, studying this response should provide insight into pathogenesis of HRV disease including exacerbations of asthma.

We chose to study the human response to HRV infection by modeling local infection with respiratory epithelial cells, the primary site of human infection, and modeling the immune response to this infection with peripheral blood mononuclear cells (PBMCs). Previously, various respiratory epithelial cells have been used to study the response to HRV infection in vitro and have shown that HRV infection induces a variety of chemokines and cytokines (eg. IL-1, IL-6, IL-8, ENA-78, eotaxin, RANTES and Gro-α) [8], [9], [10], [11], [12], [13], [14], [15]. Some of these cytokines and chemokines have been associated with asthma [5], [6]. Other investigators have also studied the response of PBMCs, or cells purified from PBMCs, to HRV infection and showed a significant increase in cytokines and chemokines such as IP-10 and IFN-α [8], [9].

In our model, we used a two-chamber trans-well tissue culture system to expose the PBMCs to the HRV-infected airway epithelial cells. With this two-chamber trans-well system, cell contact that might induce HLA-associated responses that confound our results is prevented, but virus, virus antigens and cellular proteins such as cytokines and chemokines can cross the membrane. Thus, this system allows “cross-talk” between the airway epithelial cells and the PBMCs. With this system, we can assess the response associated with different airway epithelial cells, different strains of HRV, or different sources of PBMCs. In this report, we used a constant source of airway epithelial cells, i.e. a continuous cell line, and studied differences between strains of HRV and source of PBMCs. We used production of cytokines and chemokines as an indicator of the response to the infection.

## Methods

### Ethics statement

The individuals in this manuscript have given written informed consent to participate in this study and to publish these case details. The PBMCs had been isolated from blood samples collected from four healthy adults under an Emory Institutional Review Board approved protocol (IRB #-00045690).

### Human Airway Epithelial Cells (HAEC) and Viruses

Calu-3, A549 and BEAS-2B cells were obtained from American Type Culture Collection (ATCC) and grown in minimum essential medium (MEM) supplemented with 10% fetal calf serum (FCS), 1 mM L- Glutamine, 1 mM Hepes and 1X non-essential amino acids (Calu-3 media) and incubated at 37°C under 5% CO_2_. HRV 14 and HRV 16 prototype strains were obtained from the Centers for Disease Control and Prevention (Dr. Dean Erdman). The viruses were grown in HeLa cells maintained in MEM containing 10% FCS, 1% penicillin streptomycin and 1% L-Glutamine at 35°C under 5% CO_2_. We chose to study HRV 14 (species B) and HRV 16 (species A), both major receptor group viruses, because they are commonly used for in vitro infection studies. HRV C could not be evaluated in the study because of the difficulty in cultivating the virus. HRV 14 and HRV 16 stocks were prepared by infecting monolayers of HeLa cells. The infected cells were maintained at 35°C under 5% CO_2_ until cytopathic effect (CPE) exceeded 70%. The media was collected and centrifuged briefly to remove the cellular debris, and the clear supernatant was divided into aliquots and stored at –80°C.

To determine the virus titer, Hela cells were grown in 96 well flat bottom tissue culture plates (5000 cells/well) and infected with 10-fold serial dilutions of virus in 8 replicates. The infected cells were incubated for 5 days and evaluated daily for CPE by microscopic examination. The tissue culture infectivity dose (TCID_50_) of the viruses was calculated based on the CPE according to the Reed Muench method. To assess replication of the virus in the human respiratory epithelial cell lines, 150,000 cells/well were seeded in 24 well plates and incubated at 37°C under 5% CO_2_. When the cells reached 60% confluency, they were infected with 0.5 MOI of HRV 14 and HRV 16, and the course of infection assessed at 35°C under 5% CO_2_. Two days post infection, the media was changed. Supernatants from virus infected wells and control wells were collected daily to assess levels of viral RNA. The collected media were briefly centrifuged to remove the cell debris and RNA was extracted using Qiagen RNEasy mini kit according to manufacturer’s instructions. HRV RNA was assayed by a real-time RT-PCR assay using AgPath-ID™ One-Step RT-PCR Reagents and the Applied Biosystems 7500 Fast Real-Time PCR System (Life Technologies Corporation, Carlsbad, CA) as previously described [16]. The time course of HRV 14 and HRV 16 replication in Calu-3 cells (i.e. gradual increase in virus RNA and gradual increase in CPE through 5 days post infection), was best suited to our studies and hence Calu-3 cells were used for the remaining studies. Infection was less efficient in BEAS-2B cells and CPE was extensive by 24 to 48 hrs in A549 cells (data not shown).

### HAEC and PBMC Co-culture

For the cytokine and chemokine studies, Calu-3 cells were seeded and infected as mentioned above with 0.5 MOI of infectious virus, heat-inactivated virus, and mock-infected cell control material. The inoculum was removed 2 hours later and 500 ul of Calu-3 media was added to each well. Heat-inactivated viruses were prepared by incubating the viruses at 58°C for one hour. The media was removed and fresh media was added two days post infection (p.i.). One day later, i.e. 3 days p.i., trans-well inserts (polyester trans-well inserts, 0.33 cm with 0.4u pores, Corning, Inc., Corning, NY) with one million live PBMCs (viability determined by trypan blue exclusion) were placed in the virus infected and control wells. PBMCs were purified from the blood using ficoll-histopaque density gradient centrifugation, divided into aliquots, and stored in liquid nitrogen. We chose cryopreserved cells because they are often used for PBMC studies and provide a consistent source of cells. A consistent source of cells facilitates comparisons between experiments. Media was collected from the basolateral chamber at 6, 24, and 48 hours after addition of the PBMCs. The collected media was centrifuged, stored at –80°C, and later tested by multiplex luminex assays according to the manufacturer’s instructions for FGF-Basic, IFN-γ, IL–12 (p40/p70), IL–13, RANTES, MIP–1α, MIG, MIP–1β, VEGF, IL–1β, IL–2, IL–4, IL–5, IL–6, IL–2R, MCP–1, Eotaxin, IL–8, IL–10, IL–15, IL–17, IL–1RA, GM–CSF, G–CSF, EGF, HGF, TNF-α, IL–7, IP-10, IFN-α (Human cytokine 30-plex panel, Life technologies), 6Ckine, BCA-1, CTACK, ENA-78, Eotaxin-2, Eotaxin-3, I-309, IL-16, IL-20, IL-21, IL-23, IL-28A, IL-33, LIF, MCP-2, MCP-4, MIP-1d, SCF, SDF-1A+β, TARC, TPO, TRAIL, TSLP (Human cytokine 23 plex panel, Millipore) and ENA-78, TSLP and IL-33 (Human cytokine 3 plex panel, Millipore).

### Analysis

Statistical analyses were conducted using SAS 9.2 (Cary, NC) and statistical significance was assessed at the 0.05 unless otherwise noted. Due to the small sample sizes and non-normal data, nonparametric tests were utilized. Specifically, for each cytokine, the actual data values were replaced by their rank in the dataset and the analyses were carried out on the ranked data. The median and range were used to summarize cytokine levels. We initially looked for cytokine and chemokines that had a significant increase, or decrease, with HRV infection both without and with PBMCs. In this analysis, the values for HRV-infected cells (for each HRV 14 and 16 independently) were compared to values for mock infected cells and the values for PBMCs exposed to infected cells compared to values for PBMCs exposed to mock infected cells. The cytokines that showed a significant increase or decrease (i.e. an HRV-associated response) were analyzed further using a two-factor analysis of variance model (ANOVA). With this model, we assessed the effect of the addition of PBMCs and virus type (HRV14/HRV16) on results. All models initially included a term for interactions between PBMC and virus type but this term was removed from the model if the effect was not significant. Specifically, we compared the effect of virus type on cytokine expression for different sources of PBMCs, and the effect of PBMCs on cytokine expression for each virus type. For most analyses, we compared: (1) HRV-14- without *PBM*C (N) vs. HRV-16- N *PBMCs*, (2) HRV-14- with *PBMCs* (Y) vs. HRV-16-Y *PBMC*, (3) HRV-14- N *PBMC* vs. HRV-14-Y *PBMC* and (4) HRV-16- N *PBMC* vs. HRV-16- Y *PBMC*. To control for multiple comparisons, a Bonferroni adjustment was used and statistical significance was assessed using a significance level of 0.05/4 = 0.0125.

## Results

**Figure 1:**
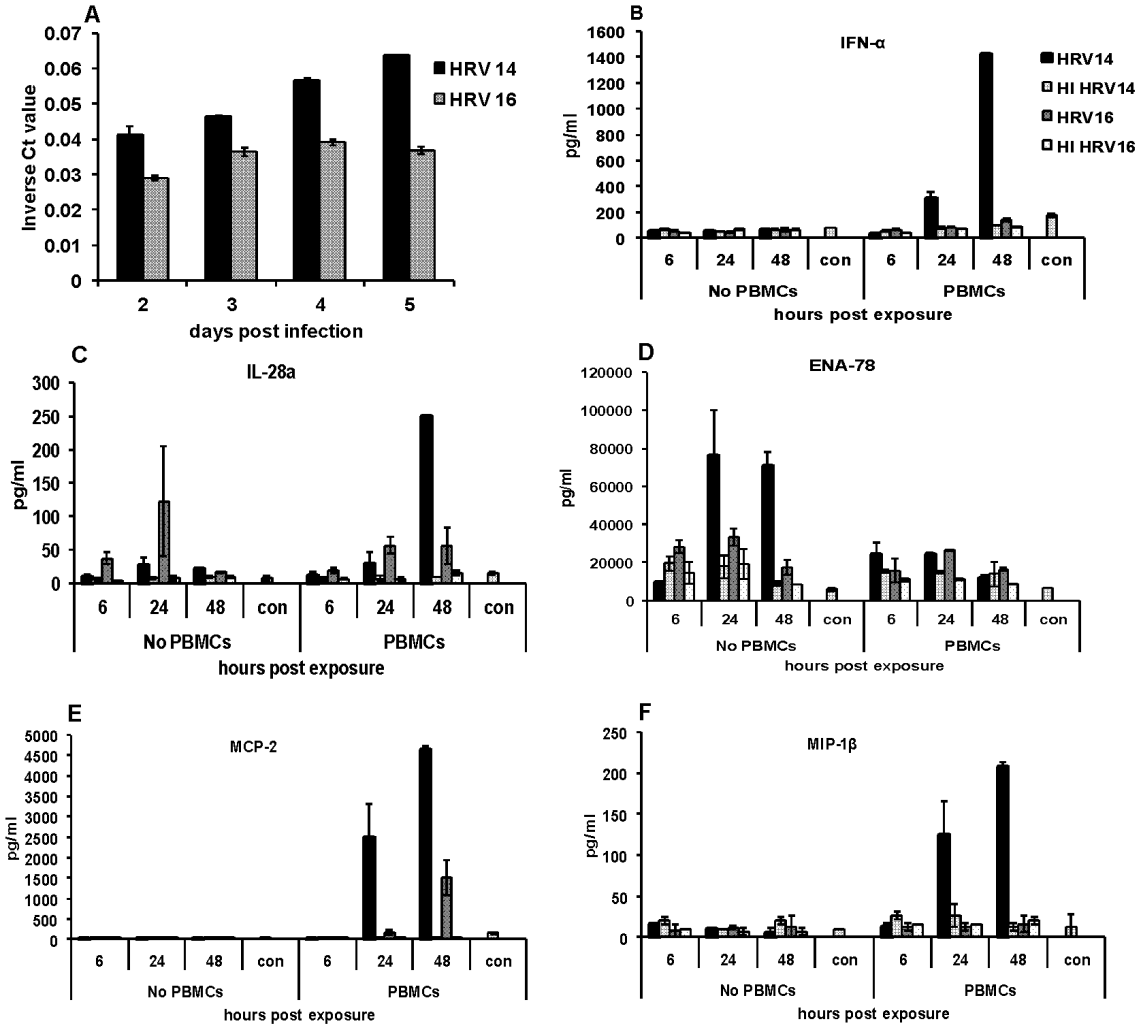
Time course of replication and cytokine production in HRV 14 and HRV 16 infected Calu-3 cells. Fig 1A. The relative amount of viral RNA detected in supernatants of Calu-3 cells infected with 0.5 MOI of HRV 14 or HRV 16 in real time (RT) PCR and represented as inverse cycle threshold (Ct) values. Ct levels reflect the number of cycles required to exceed the background level; inverse Ct levels (1/Ct) are proportional to the amount of target nucleic acid in the sample. RT-PCR underwent 40 cycles of amplification. The data are represented as average ± SD from a representative experiment. Fig 1B, 1C, 1D, 1E and 1F . **Levels of cytokines (pg/ml) in the supernatant of Calu-3 cells** infected with HRV 14 or HRV 16, heat inactivated (HI) viruses and uninfected control (con) cells with or without PBMCs. The 6, 24 and 48 hour time points represent hours after the time inserts with PBMCs were added to the Calu-3 cells. Data are average ± SD from duplicate wells for one experiment that is representative of the four experiments.

The replication kinetics based on real-time RT-PCR Ct values varied between the viruses (Fig 1A) with HRV 14 growing more efficiently than HRV 16. Infectivity titrations on supernatants of the Calu-3 infected cells, similar to results for RT-PCR, showed gradual increase in infectious virus over time and more efficient replication for HRV 14 (data not shown).

As noted in earlier studies, a number of chemokines and cytokines were induced by HRV infection of airway epithelial cells. Not previously described, however, are the types of differences in responses between HRV 14 and HRV 16 and the impact of adding PBMCs on responses to infection. Initially we looked at the cytokine/chemokine secretion at 1, 2, 3, 4, and 5 days p.i without the addition of PBMCs. We saw substantially lower levels of cytokines and chemokines (often not significantly above mock infected levels) at 1 and 2 days p.i (data not shown) and consistent increases above mock infected levels at 3, 4, and 5 days p.i. Peak levels were often achieved at 4 or 5 days post infection. Hence, we focused our analysis on days 3–5 p.i. Virus or cellular proteins in the inoculum should not have been present at 3 days p.i. since the inoculum was removed 2 hrs post infection and the media was changed 2 days p.i. Since the levels of cytokines/chemokines induced by heat inactivated (HI) HRV control or mock-infected control inoculum were similar, we used mock-infected control for most comparisons. HRV-associated change in the levels, i.e. difference between HRV-infected compared to mock infected cells at 3-5 days p.i was noted for IL-6, IL-15, MIP-1β, IL-28A, interferon gamma-induced protein 10 (IP-10 or CXCL10), epithelial neutrophil activation protein-78 (ENA-78), and basic fibroblast growth factor (FGF-Basic) without PBMCs (Table 1). In addition, differences in responses induced by HRV 14 compared to HRV 16 infected cells were evident for ENA-78, FGF-Basic, IL-6, IP-10, MIP-1β, IFN-α, MCP-2 and IL-28A, (Table 1).

**Table 1:** HRV 14 and HRV 16-associated responses in Calu-3 cells with or without PBMCs.

Cytokine/Chemokine	Median (Range in pg/ml)		
	Virus	Without PBMC	With PBMC
**FGF-Basic**	HRV 14	68.4 (49.9–90.3)*	52.1 (42.0–64.8)*
	HRV 16	167.8 (106–201)*	146.1 (126–181)*
	Uninfected	18 (9.8–23.6)	20.4 (17.5–32.3)
**IL-15**	HRV 14	528 (474–564)*	584 (398–967)*
	HRV 16	384 (108–796)	472 (337–737)
	Uninfected	210 (100–417)	226 (100–359)
**IP-10**	HRV 14	1559 (1006–1765)*	885 (752–900)*
	HRV 16	355 (245–462)*	434 (382–553)*
	Uninfected	52.4 (24.4–178)	95.8 (16.2 (172)
**IL-6**	HRV 14	1731 (1488–1974)*	897 (817–1038)*
	HRV 16	4112 (3499–4525)*	1159 (780–1482)*
	Uninfected	690 (368–817)	548 (249–734)
**MIP-1β**	HRV 14	7.0 (5.6–8.3)**	353 (208–438)*
	HRV 16	27.5 (11.9–92.9)	40.8 (16.4–77.6)
	Uninfected	14.6 (9.6–41.2)	31.5 (9.8–128)
**IFN-α**	HRV 14	61.2 (29.2–101)	1447 (1303–2375)*
	HRV 16	66.8 (30.1–130)	146 (140–212)
	Uninfected	74.3 (40–90.5)	154 (110–198)
**MCP-2**	HRV 14	8.4 (5.9–11.9)	4308 (3883–4902)*
	HRV 16	5.9 (5.2–15.2)	1511 (1012–2338)*
	Uninfected	8.1 (6.3–10.1)	170 (10.4–208)
**IL-28A**	HRV 14	21.1 (12.3–22.8)	236 (196.7–250.9)*
	HRV 16	32.9 (16.5–55.5)	51.7 (11.4–58.2)
	Uninfected	8.2 (6.1–20.1)	18.5 (15.5–28.6)
**ENA-78**	HRV 14	73733 (68961–85867)*	13862 (11461–16022)*
	HRV 16	17609 (14076–21401)*	16752 (11048–19686)*
	Uninfected	8149 (5914–10656)	6941 (5086–10275)

Shown are the median values of cytokines and chemokines (pg/ml) produced in response to HRV 14, HRV 16 and uninfected control infections at 48 hours after the time inserts with PBMCs were added (n = 4). Significant differences in cytokine/chemokine levels for HRV 14 and HRV 16 infected cells when compared to uninfected controls were calculated using Wilcoxon rank sum tests. * indicates values for HRV 14 or HRV 16 that are significantly, P<0.05, above values for the uninfected control. ** indicates that the level of MIP-1β in HRV 14 infected Calu-3 cells without PBMCs is significantly lower than the uninfected controls. * P<0.05.

The addition of PBMCs to the infected cells affected results for some but not all cytokines. For the cytokines FGF basic and IL-15 the addition of PBMCs did not significantly change the levels (Table 1) and for IL-6 the addition of PBMCs did not affect the relative differences between the levels associated with HRV 14 and HRV 16 infection. Hence PBMCs were removed from ANOVA model for these three cytokines/chemokines. HRV 16 produced higher levels of FGF-basic compared to HRV 14 [(median values -157.2 pg/ml vs.57.1 pg/ml) (p<0.001)]. There was no significant difference in IL-15 levels between the two viruses [(530.7 pg/ml vs. 425.6 pg/ml) (p = 0.228)]. HRV 16 produced higher levels of IL-6 compared to the HRV-14 virus [(2490 pg/ml vs. 1263 pg/ml) (p = 0.018)] and the addition of PBMCs significantly decreased IL-6 expression for both viruses (p<0.001). For the other cytokines and chemokines the PBMCs did affect results and PBMCs were included in the model (Table 2). HRV 14 produced higher levels of IP-10 compared to HRV 16 for both with and without PBMCs (p = 0.003 and p<0.001, respectively) (Table 2). The addition of PBMCs significantly decreased IP-10 expression in HRV 14 (p = 0.012); but not in HRV 16 infections (p = 0.163) (Table 2). Without PBMCs, HRV 16 produced significantly higher levels of MIP-1β compared to HRV 14 (p = 0.003); however, when PBMCs were added, HRV 14 produced significantly higher levels of MIP-1β compared to HRV 16 (p = 0.003). The addition of PBMCs significantly increased MIP-1β expression in HRV 14 infected cells (p<0.001), whereas PBMCs had no effect on MIP-1β expression in the HRV 16 virus (p = 0.502) (Table 2, Fig 1F).

**Table 2:** Virus-associated cytokine/chemokine (pg/ml) responses with and without PBMCs in Calu-3 infected cells (n = 4).

Cytokine/Chemokine	Medain (Range in pg/ml)			P value		
	PBMC	HRV 14	HRV 16	HRV14 vs HRV16	HRV14 (N) vs HRV14(Y)	HRV16 (N) vs HRV16(Y)
IP-10	N	1559 (1006-1765)	355 (245-462)	<0.001*	0.012*	0.163
	Y	885 (752-900)	434 (382-553)	0.003*		
MIP-1β	N	7.0 (5.6-8.3)	27.5 (11.9-92.9)	0.003*	<0.001*	0.502
	Y	353 (209-439)	40.8 (16.4-77.6)	0.003*		
IFN-α	N	61.2 (29.2-101)	66.8 (30.1-131)	0.502	<0.001*	0.003*
	Y	1447 (1304-2375)	146 (141-212)	0.017		
IL-28A	N	21.1 (12.3-22.8	33.0 (16.5-55.5)	0.232	0.001*	0.578
	Y	236 (197-251)	52 (11.4-58)	0.014		
ENA-78	N	73733 (68961-85868)	17609 (14076-21402)	0.017	<0.001*	0.352
	Y	13862 (11461-16022)	16752 (11048-19686)	0.208		
MCP-2	N	8.4 (5.9-11.8)	5.9 (5.2-15.2)	0.502	<0.001*	<0.001*
	Y	4308 (3883-4902)	1511 (1012-2338)	0.016*		

**“**N” indicates No PBMC exposure and “Y” indicates PBMC exposure. Two-factor ANOVA models were used. Four pairwise comparisons were made between HRV 14 and HRV 16 with and without PBMCs. * indicates statistical significance at 0.0125 level.

Without PBMCs, there was no difference in IFN-α expression by HRV serotype (p = 0.502) (Table 2, Fig 1B). After the addition of PBMCs, HRV 14 produced higher levels of IFN-α (Table 2, Fig 1B); however, not significantly so (p-value of 0.017, the Bonferroni adjusted significance level is 0.0125). For both HRV 14 and HRV 16, the addition of PBMCs significantly increased IFN-α expression (p<0.001 and p = 0.003, respectively) (Table 2). Without the addition of PBMCs, there was no difference in IL-28A expression between HRV 14 and HRV 16 viruses (p = 0.232) (Table 2) 5 days p.i. After the addition of PBMCs, HRV 14 produced higher levels of IL-28A compared to HRV 16, but not significantly so (p-value of 0.014) (Table 2). The addition of PBMCs significantly increased IL-28A expression with HRV 14 (p = 0.001), but did not change expression of IL-28A in the HRV 16 virus (p = 0.578) (Table 2, Fig 1C). Without the addition of PBMCs, HRV 14 produced higher levels of ENA-78 (p = 0.017), while with the addition of PBMCs, there was no difference in levels between the HRV 14 and HRV 16 (p = 0.208). Adding PBMCs significantly decreased ENA-78 expression in HRV 14 infected cells (p<0.001), but did not affect expression in HRV 16 infected cells (p = 0.352) (Table 2, Fig 1D). Finally, there was no significant difference in MCP-2 levels between HRV 14 and HRV 16 without PBMCs (p = 0.502) but with PBMCs, levels of MCP-2 were higher in HRV 14 infected cells but not significantly so (p = 0.0169) (Table 2, Fig 1E).

While most of the HRV-associated responses were evident at all points with greatest differences at the 5 days p.i., some were evident at some but not all time points (Fig 1B, 1C, 1D and 1F). For example, a much larger increase in IL-28A was detected for HRV 16 without PBMCs at 4 days p.i. than at 5 days p.i. (Fig 1C). We also noted donor to donor differences in the expression of some cytokines like MCP-1, HGF, IL-1RA and RANTES not included in the analysis above. For these cytokines, increases in levels compared to mock-infected cells were consistent with an HRV-associated response for some but not all PBMCs as illustrated in Table 3. These results suggest an HRV 16-associated response for MCP-1 and HRV 14-associated response for HGF for PBMC 2 and PBMC 3. For IL-1RA, the values are suggestive of an HRV 14-associated response for PMBC 2 and PBMC 3 and HRV 16-associated response for PBMC 3. For RANTES, the values are suggestive of an HRV 14-associated response for PBMC 1 and PBMC 2 and an HRV 16-associated response for PBMC 4. These types of differences in responses between sources of PBMCs are being further characterized. Next, to assess the likelihood that experimental variability might explain some of the differences detected, we analyzed responses in two separate experiments with PBMCs from the same blood sample from the same donor. As noted in Table 4, the results between experiments from the same PBMCs were consistent and experiment to experiment variation does not explain the extent of differences between HRV strains or PBMCs. Next, to determine if the extent of virus replication affected cytokine and chemokine levels and might explain the differences that we saw between HRV 14 and HRV 16, we used a higher dose of HRV 16 (2.5 or 5 MOI) which resulted in similar levels of HRV RNA between HRV 14 (at MOI of 0.5 or 1) and HRV 16 at the 24 hr and 48 hr time points. Although the higher MOI for HRV 16 gave a slight increase in some cytokines, e.g. IFN-α and MIP-1β (data not shown), it did not affect the significance of differences between chemokine and cytokines levels for HRV 14- and HRV 16- infected cells. Finally, to check if factors in the virus inoculum affected our results, we purified the viruses through 20% sucrose cushion gradient and infected Calu-3 cells with the purified viruses. We saw a similar pattern and level of cytokine and chemokine responses between the purified and the unpurified inoculums. Overall, the levels of each cytokine and chemokines secreted by HRV 14, HRV 16 or uninfected control infections without PBMCs, were within the range as given in Table 1 suggesting that non-virus factors present in the unpurified inoculum did not affect our results.

**Table 3:** Donor to donor difference in some cytokines secreted in HRV 14, HRV 16 and uninfected Calu-3 cells.

Cytokine/Chemokine	PBMC 1			PBMC 2			PBMC 3			PBMC 4		
	HRV 14	HRV 16	Uninf	HRV 14	HRV 16	Uninf	HRV 14	HRV 16	Uninf	HRV 14	HRV 16	Uninf
MCP-1	34307	154858	31706	6100	2939	6100	33717	318876	32996	8047	7308	3984
HGF	ND	ND	ND	159	86	69	370	167	95	402	348	666
IL-1RA	1140	710	677	1702	985	543	570	585	233	924	916	1172
RANTES	346	58	135	175	56	65	498	525	418	13	56	5

Shown are the values of cytokine/chemokines (pg/ml) produced from four different PBMCs and data are for 48 hours after the time PBMCs were added to the Calu-3 cells. ND is not detected. Note values for HRV infected compared to uninfected cells suggestive of an HRV 16-associated response for MCP-1 and HRV 14-associated response for HGF for PBMC 2 and PBMC 3. For IL-1RA, the values are suggestive of an HVR 14-associated response for PMBC 2 and PBMC 3 and HRV 16-associated response for PBMC 3. For RANTES, the values are suggestive of an HRV 14-associated response for PBMC 1 and PBMC 2 and an HRV 16-associated response for PBMC 4.

**Table 4:** Experiment to experiment variation in cytokine/chemokine responses of PBMCs from same donor to HRV-infected Calu-3 cells.

Cytokine/Chemokine	HRV 14		HRV 16		Uninfected	
	Exp 1	Exp 2	Exp 1	Exp 2	Exp 1	Exp 2
**FGF-Basic**	65	48	127	168	22	32
**IL-15**	530	483	390	356	150	127
**IP-10**	900	827	454	366	45	39
**IL-6**	900	936	1288	1383	246	307
**MIP-1β**	209	298	17	25	13	22
**IFN-α**	1433	1366	146	207	134	196
**MCP-2**	4644	4397	1439	1563	27	13
**IL-28A**	361	220	56	48	16	20
**ENA-78**	12087	11932	16538	12498	10255	6632

Shown are the values of cytokine/chemokines (pg/ml) produced from the same source of PBMCs and data are for 48 hours after the time PBMCs were added to the Calu-3 cells.

## Discussion

Infection of respiratory epithelial cells with HRV as well as other respiratory viruses has been shown to induce various chemokines and cytokines that likely participate in the inflammatory or immune response to infection. In this report, we describe use of a two-chamber trans-well system to expose PBMCs to HRV-infected respiratory epithelial cells to model the immune response to this infection. With this system, we looked for changes to immune responses when the infecting virus or the source of immune cells was varied. Our data show that both virus and source of PBMCs can affect the immune response as indicated by changes in secreted cytokines.

A variety of cytokines and chemokines were induced by both HRV 14 and/or HRV 16 infection that include FGF-Basic, chemokines and cytokines like IL-6, IL-15, MCP-2, IP-10, MIP-1β, type I IFN-α, type III IFN-λ2 (IL-28A), and ENA-78. Some of these responses have been previously noted [8], [9], [17]. We did not see increases for all the cytokines or chemokines previously reported to be induced by HRV infection. For example, we did not see increases in IL-1, IL-8, GM-CSF, and eotaxin. The reason for these differences is not clear, but could result from differences in the HRVs studied, the cells used for the infection, or other differences in methods.

The addition of PBMCs to the HRV-infected Calu-3 cells had substantial effect on the cytokine and chemokine responses. These changes include increases in levels of IL-28A, IFN-α, MCP-2 and MIP-1β for one or both viruses and decreases in levels of IP-10, IL-6, and ENA-78 for one or both viruses. Korpi-Steiner et al. also looked at the interaction of HRV 16-infected airway epithelial cells and human immune cells. They studied monocytes purified from PBMCs, and used co-culture or supernatant from monocytes exposed to HRV 16 to demonstrate a monocyte induced increase in CXCL10 (IP-10) production by infected airway epithelial cells [8]. Another group noted an increase in IL-6, IL-8 and RANTES production with the addition of media from HRV exposed PBMCs to HRV-infected airway epithelial cells [18]. Stokes et al found that addition of monocytes to HRV 1B-infected BEAS-2B cells did not enhance production of IL-8 or RANTES though IL-8 production was increased when infected cells were also treated with LPS [19]. The interaction between airway epithelial cells and human immune cells during HRV infection is likely important to the disease process. The cytokines and chemokines induced by HRV infection of Calu-3 cells and/or Calu-3 cells plus PBMCs are associated with a range of functions including activation of immune cells that are implicated in allergic responses, cell proliferation, anti-viral activity, and pro-inflammatory and anti-inflammatory functions.

Several findings suggest directions for future study. One interesting finding is the difference in induction of ENA 78 between HRV 14 and HRV 16. ENA-78 has been reported to be induced by HRV infection of BEAS-2B cells and suggested to have a role in allergy and airway inflammation [18], [20]. In the present study, the addition of PBMCs to HRV 14-infected cells down regulated ENA-78 production but did not affect the level of ENA-78 produced by HRV 16-infected cells. Similarly, the addition of PBMCs to HRV 14-infected cells led to increased levels of IFN-α, MIP-1β and IL-28A and MCP-2 levels were increased in both HRV 14 and HRV 16 infected cells when exposed to PBMCs. Previous studies show that increases in IFN-α levels are associated with decreased levels of ENA-78 [21]. The reduction in the levels of some cytokines and chemokines after PBMC exposure is not surprising since PBMCs do play an important role in regulation of immune responses by a number of mechanisms including release of mediators that regulates cytokine or chemokine degradation or receptor mediated consumption of cytokines [22], [23]. This in vitro model provides a way to study the effect of production of one cytokine on production of other as well as HRV strain differences in cytokine and chemokine production.

Strain differences in response to HRV infection are consistent with earlier observations. Wark and colleagues [24] measured release of IL-6, IP-10, IFN-β and IFN-γ by HRV-infected primary bronchial epithelial cells and noted higher levels induced by recent clinical isolates compared to laboratory passaged strains and differences in levels between cells infected with HRVs from the major and minor receptor groups. They also noted differences in levels produced by infected primary bronchial epithelial cells from asthmatic compared to non-asthmatic patients. With their system, they did not detected differences in levels of cytokines or chemokines induced by HRV 14 and HRV 16 [24]. Species differences in the way the HRV 2A protease alters cell functions has been recently reported and suggests one possible mechanism for the differences we noted [25]. Epidemiologic studies have shown HRV species differences in associations with asthma exacerbations with species A HRVs showing stronger associations than species B HRVs [26]. HRV 14 is a species B virus and HRV 16 is a species A virus. The HRV-induced responses seen in our in vitro model provides another way to study the impact of strain variation on disease outcome.

Responses to HRV infection by PBMCs also provides a promising way to study patient differences that may contribute to disease, e.g. exacerbations of asthma. Others have noted and studied cytokine or chemokine responses during HRV infection in respiratory specimens from patients with and without asthma. For example, in studies of respiratory secretions, Gern et al reported that experimental infection of allergic individuals with HRV 16 resulted in increased levels of IL-8 and granulocyte colony stimulating factor (GCSF) [27], Miller et al found an association between increased levels of Type III IFN-λ and wheezing with HRV infection in patients with asthma [28] and Garcia et al noted lower levels of MCP-1 and IL-1Ra associated with increased severity of HRV infection [29]. Groups have also looked at cytokines and chemokines induced by exposing immune cells from patients with asthma to HRVs. For example, Forbes et al noted lower levels of type I and III IFN in HRV43 and 1B exposed PBMCs from pregnant women with asthma [30], Iikura et al. found lower levels of IFN-α and inflammatory and other cytokines in PBMCs from patients with asthma exposed to HRV 14 [31] and Sykes et al. found decreased type I IFN responses to HRV 16 exposed BAL cells from patients with asthma [32].

In conclusion, the results of this study show that differences in the virus and host can lead to changes in the expression of various cytokines produced by human airway epithelial cells and/or PBMCs responding to HRV infection. These differences in cytokine and chemokine expression may be associated with and help explain differences in disease such as previously noted differences between infections with species A and B HRVs or between infections in children with and without asthma. This model provides one way to study the basis for these differences and pathogenesis of HRV disease.
